# Secondary Metabolite Profiling of *Curcuma* Species Grown at Different Locations Using GC/TOF and UPLC/Q-TOF MS

**DOI:** 10.3390/molecules19079535

**Published:** 2014-07-04

**Authors:** Jueun Lee, Youngae Jung, Jeoung-Hwa Shin, Ho Kyoung Kim, Byeong Cheol Moon, Do Hyun Ryu, Geum-Sook Hwang

**Affiliations:** 1 Integrated Metabolomics Research Group, Western Seoul Center, Korea Basic Science Institute, Seoul 120-140, Korea; E-Mails: lje3080@kbsi.re.kr (J.L.); jya0819@kbsi.re.kr (Y.J.); jhshin01@kbsi.re.kr (J.-H.S.); 2 Department of Chemistry, Sungkyunkwan University, Suwon 440-746, Korea; 3 Basic Herbal Medicine Research Group, Herbal Medicine Research Division, Korea Institute of Oriental Medicine, Daejeon 305-811, Korea; E-Mails: hkkim@kiom.re.kr (H.K.K.); bcmoon@kiom.re.kr (B.C.M.); 4 Herbal Material Management Team, Herbal Medicine Research Division, Korea Institute of Oriental Medicine, Daejeon 305-811, Korea; 5 Graduate School of Analytical Science and Technology, Chungnam National University, Daejeon 305-764, Korea

**Keywords:** *Curcuma aromatica*, *Curcuma longa*, *Zngiberaceae*, metabolite profiling, secondary metabolites, GC/TOF MS, UPLC/Q-TOF MS

## Abstract

*Curcuma*, a genus of rhizomatous herbaceous species, has been used as a spice, traditional medicine, and natural dye. In this study, the metabolite profile of *Curcuma* extracts was determined using gas chromatography-time of flight mass spectrometry (GC/TOF MS) and ultrahigh-performance liquid chromatography–quadrupole time-of-flight mass spectrometry (UPLC/Q-TOF MS) to characterize differences between *Curcuma aromatica* and *Curcuma longa* grown on the Jeju-do or Jin-do islands, South Korea. Previous studies have performed primary metabolite profiling of *Curcuma* species grown in different regions using NMR-based metabolomics. This study focused on profiling of secondary metabolites from the hexane extract of *Curcuma* species. Principal component analysis (PCA) and partial least-squares discriminant analysis (PLS-DA) plots showed significant differences between the *C. aromatica* and *C. longa* metabolite profiles, whereas geographical location had little effect. A *t*-test was performed to identify statistically significant metabolites, such as terpenoids. Additionally, targeted profiling using UPLC/Q-TOF MS showed that the concentration of curcuminoids differed depending on the plant origin. Based on these results, a combination of GC- and LC-MS allowed us to analyze curcuminoids and terpenoids, the typical bioactive compounds of *Curcuma*, which can be used to discriminate *Curcuma* samples according to species or geographical origin.

## 1. Introduction

*Curcuma* is a genus comprised of ~70 known species in the plant family Zingiberaceae and has been historically used as a spice, medicine, and ceremonial dye [1]. Recently, this genus has been investigated for possible medicinal benefits, including antioxidant and anti-inflammation properties [2,3].

Curcuminoids and terpenoids are the major *Curcuma* bioactive compounds. Curcuminoids are vivid yellow natural phenols with antioxidant, anti-inflammatory and chemotherapeutic activities, and include curcumin (**1**) and its derivatives, such as demethoxycurcumin (**2**) and bisdemethoxycurcumin (**3**) [4,5]. Terpenoids are a diverse class of phytochemicals including aromatic, non-aromatic, volatile and non-volatile constituents that play a key role in traditional herbal remedies and are being investigated for antibacterial, antineoplastic, and other pharmaceutical potentials [6,7,8].

In metabolomics, mass spectrometry (MS) combined with chromatography is considered a keystone analytical technology [9] and is commonly used due to its large dynamic range, reproducibility, and ability to analyze complex biological samples [10]. This coupled analytical technique has been used to discriminate between plant species [11] and to characterize the biochemical behavior of plants exposed to various environmental situations, including growth conditions, stress, or seasonal changes [12,13]. Raven *et al.* investigated specific phytochemicals that were either genus or species specific [14], and found that these chemical components were affected by environmental factors such as temperature, precipitation, soil conditions, and seasonality [15]. GC/TOF MS technology offers high mass resolution, high mass accuracy and fast scan speeds, and has been applied recently in metabolomics for large-scale metabolite profiling of plant materials [16]. In addition, the combination of GC-MS and LC-MS has advantages for the analysis of compound classes over a range of polarity and relative molecular mass. The high polar components are profitable for LC-MS analysis whereas GC-MS are generally used to analyze non-polar components such as essential oils [17]. This dominant characteristics allow to analyze diverse components in crude extracts of plant materials and obtain more broad and comprehensive metabolite profiles [18,19].

In this study, MS-based metabolite profiling was used to investigate secondary metabolites that could discriminate between *Curcuma longa* and *Curcuma aromatica* grown in different locations (Jeju-do and Jin-do). *C. aromatica* and *C. longa* are representative *Curcuma* species grown in Korea, and both have long been cultivated in the southern islands of Jeju-do and Jin-do.

The abundant primary components of *C. aromatica* and *C. longa* grown in different regions were investigated using NMR-based metabolomics [20]. The same species was subsequently grown in the same regions and collected at the same time, but subjected to different extraction methods and analytical platforms. This present study reports the secondary metabolites of *C. aromatica* and *C. longa* grown in two locations using the combination of liquid chromatography (LC)- and gas chromatography (GC)-mass spectrometry. The typical bioactive compounds of *Curcuma* are curcuminoids and terpenoids, and these secondary metabolites can be analyzed using LC-MS and GC-MS, respectively. Furthermore, global and targeted metabolite profiling was used to identify possible species- and origin-specific markers.

## 2. Results and Discussion

Previous studies on *Curcuma* using MS were qualitative analyses [21,22] focusing on specific secondary metabolites such as curcuminoids [23,24] and essential oils [25,26]. To our knowledge, no qualitative and quantitative analysis of major secondary metabolites in *Curcuma* extracts has been performed. Moreover, we identified specific markers of *Curcuma* species using metabolic profiling and target analysis.

### 2.1. GC/TOF MS Analysis of Non-Polar Extracts in Curcuma Extracts

**Figure 1:**
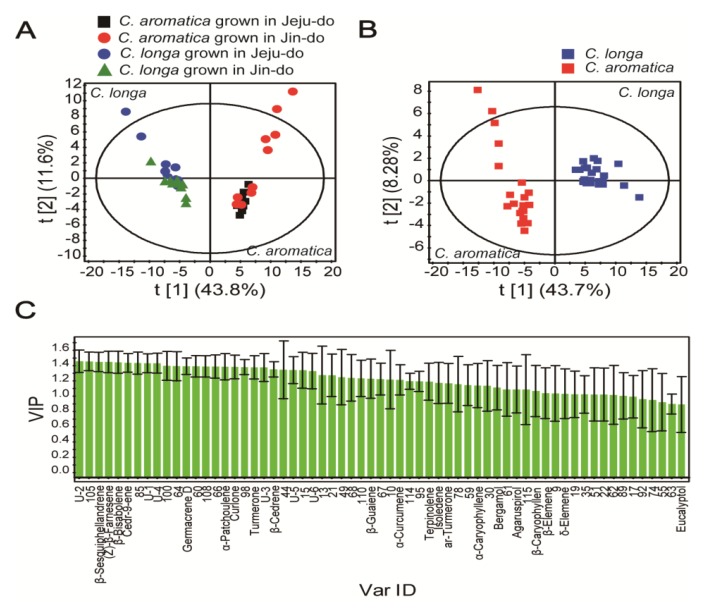
PCA score plot (**A**) derived from the GC/TOF MS spectra of non-polar *Curcuma* extracts from the two *Curcuma* species (*C. longa* and *C. aromatica*) grown at different locations: R^2^X = 0.623 and Q^2^ = 0.406; PLS-DA score (**B**) and VIP plots (**C**) derived from GC/TOF MS spectra of non-polar *Curcuma* extracts from the two *Curcuma* species grown at different locations: R^2^X = 0.584, R^2^Y = 0.982 and Q^2^ = 0.949. The ellipse represents the 95% confidence region for Hostelling’s *T*^2^. The y-axis of the VIP plot denotes the values of first variable importance in the projection in the VIP plot. U-1 to U-6 refer to unidentified compounds **1** to **6**, respectively.

Based on spectral similarities and retention time-shift corrections, the integrated signal of the base peak ion was generated using the MarkerLynx software (Waters, Manchester, UK) and was subsequently used to generate multivariate statistics. A total of 121 features was obtained from GC/TOF MS spectra. Terpenoids (non-polar constituents) were included in *Curcuma* [27] and extracted using *n*-hexane [21,28]. Therefore, terpenoids were major components of non-polar extracts of *Curcuma*. To provide comparative interpretations and to visualize metabolic differences between *Curcuma* extracts grown in different areas, PCA and PLS-DA were applied to the GC/TOF MS spectrum data set. PCA score plots of GC/TOF MS spectra of *Curcuma* showed that the separation between *C. longa* and *C. aromatica* was clearer than between Jeju-do and Jin-do (Figure 1A). In addition, only t [1] (43.8% of variation) among the three t-axes generated using the PCA model showed significant differences between the two *Curcuma* species (*p* < 0.05). These results suggested that the species difference was larger than the geographical difference. This seems reasonable given that non-polar compounds are secondary metabolites and are not directly involved in the normal growth, development, or reproduction of an organism [29], and are restricted to a narrow set of compounds within a phylogenetic group [30]. Unique features were observed in the two *Curcuma* species (20 features for *C. aromatica* and 58 for *C. longa*), and a larger number of metabolites were present in *C. longa* than *C. aromatica*. These phenotypic variations in plant secondary chemicals arise from a combination of genetic, developmental and environmental sources [31]. Therefore, a greater number of rhizome essential oils are present in *C. longa* than *C. aromatica*, and so *C. longa* may have a stronger aromatic taste and more therapeutic potential than *C. aromatica*. Previous studies reported that non-polar compounds extracted from the rhizome of *Curcuma* species possessed antioxidant activities, and *C. longa* showed greater effectiveness than *C. aromatica* [32]. In addition, Ritwiz *et al.* revealed that among five species of *Curcuma* containing *C. aromatica*, *C. longa* had the highest antioxidant and antibacterial activities of leaf extract [33].

To identify the *Curcuma* metabolites responsible for species differences, the PLS-DA score (Figure 1B) and variable importance in the projection (VIP) plot (Figure 1C) were generated. The PLS-DA model applied to the two *Curcuma* species showed a significant separation along the t [1] axis. R^2^X is the percentage of all GC/TOF MS response variables explained by the model. R^2^Y is the percentage of all observations or sample variables explained by the model. Q^2^ is the percentage of all observation or sample variables predicted by the model. The PLS-DA model showed an acceptable value of R^2^X (0.584), R^2^Y (0.982) and Q^2^ (0.949). Based on these results, 58.4% of response variables and 98.2% of observations were confidently explained to describe the difference between two *Curcuma* species. Moreover, a total of 94.9% of observations was predicted using this model. To validate PLS-DA model, permutation test using 100 random permutations was conducted (Figure S1). Therefore, the PLS-DA model can be used to discriminate and identify the two *Curcuma* species. VIP values were used to reflect the importance of variables in the model with respect to correlation with all responses and projections. The VIP reflects the influence of every term in the matrix *X* on the *Y* variables, where VIP values of ±1.0 are considered the most relevant for explaining *Y* [34].

Inspection of the VIP plot related to separation showed that 63 features had a VIP value >0.9, suggesting that changes in the concentration of these metabolites are significant (Figure 1C). The x-axis shows the 63 features labeled by chemical name, or arbitrarily assigned peak number for compounds that were not identified.

**Figure 2:**
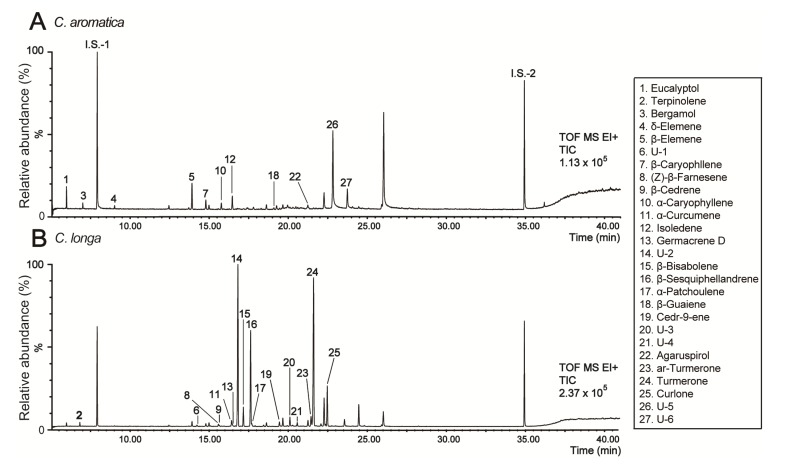
Representative GC/TOF MS chromatographic profiles of non-polar metabolites from a hexane extract of *Curcuma* species: **1**, Eucalyptol; **2**, Terpinolene; **3**, Bergamol; **4**, δ-Elemene; **5**, β-Elemene; **6**, U-1; **7**, β-Caryophyllene; **8**, (Z)-β-Farnesene; **9**, β-Cedrene; **10**, α-Caryophyllene; **11**, α-Curcumene; **12**, Isoledene; **13**, Germacrene D; **14**, U-2; **15**, β-Bisabolene; **16**, β-Sesquiphellandrene; **17**, α-Patchoulene; **18**, β-Guaiene; **19**, Cedr-9-ene; **20**, U-3; **21**, U-4; **22**, Agaruspirol; **23**, ar-Turmerone; **24**, Turmerone; **25**, Curlone; **26**, U-5; **27**, U-6; The labeled chemicals were identified with p-values less than 0.05 and VIP values greater than 0.9. U-1 to U-6 refer to unidentified compounds **1** to **6**, respectively.

**Figure 3:**
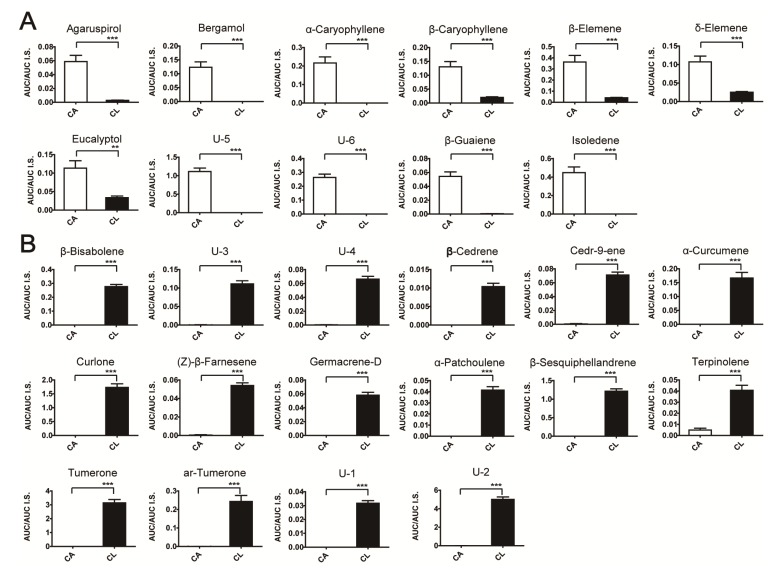
Metabolites detected in non-polar extracts of *C. aromatica* (**A**) and *C. longa* (**B**) at higher concentrations based on the *t*-test (*p* < 0.05) using GC/TOF MS. CA and CL indicate *C. aromatica* and *C. longa*, respectively. Error bars indicate ± standard errors. *, **, and *** represent *p* < 0.05, *p* < 0.01, and *p* < 0.001, respectively, as determined by Welch’s *t*-test. U-1 to U-6 refer to unidentified compounds **1** to **6**, respectively.

A statistical analysis was performed to investigate whether differences in metabolite concentrations were significant. Welch’s *t*-test was performed to examine significant differences in normalized peak intensities using methyl nonadecanoic acid (internal standard) between the two *Curcuma* species. A total of 96 features were identified as significant among the 121 features (*p* < 0.05). A total of 63 features with a VIP value above 0.9 and p-value less than 0.05 was selected and identified by searching the NIST mass spectral library. Several metabolites were verified using commercially available compounds and by performing analysis in chemical ionization mode. A total of 27 metabolites was identified, and of these, 26 metabolites (excluding eucalyptol) showed significant differences between groups after a Bonferroni correction for a p-value of 0.00041 (0.05/121) and a false discovery rate (FDR) <0.05 (Supplementary data, Table S1). In particular, the MS patterns of 27 metabolites showed at least 70% similarity to the library spectra (Table 1). Superscript a, b, and c represent similarities of 90%, 80%, and 70% with the library spectra, respectively. A total of six metabolites was labeled as unidentified in Table 1, and may be isomers or specific metabolites based on the fragmentation pattern: unidentified 1 and 2, α-zingiberene; unidentified 3 and 4, cedrene; unidentified 5 and 6, curdione. Typical total ion chromatograms of two *Curcuma* species (*C. aromatica* and *C. longa* grown in Jeju-do) from the nonpolar phase are shown in Figure 2. A comparison of *C. aromatica* (Figure 2A) and *C. longa* (Figure 2B) TICs showed the significantly different metabolites between the two *Curcuma* species. Specially, the bergamol (peak 3), α-caryophyllene (peak 10), isoledene (peak 12), unidentified 5 (peak 26) and unidentified 6 (peak 27) were found only in *C. aromatica*. In contrast, some volatile metabolites, such as (Z)-β-farnesene (peak 8), β-cedrene (peak 9), α-curcumene (peak 11), germacrene D (peak 13), β-bisabolene (peak 15), β-sesquiphellandrene (peak 16), α-patchoulene (peak 17), cedr-9ene (peak 19), aromatic (ar)-turmerone (peak 23), turmerone (peak 24), curlone (peak 25), unidentified 1 (peak 6), unidentified 2 (peak 14), unidentified 3 (peak 20), and unidentified 4 (peak 21) were detected only in *C. longa*. In total, 27 metabolites were quantified and shown in Figure 3. Peak intensities (analyte/internal standard) of 0 indicate that signals were below the detection limits or the metabolites were not detected in the *Curcuma* species. The metabolites detected in *C. aromatica* at higher concentrations included agaruspirol, bergamol, α-caryophyllene, β-caryophyllene, β-elemene, δ-elemene, unidentified 7, unidentified 8, β-guaiene, and isoledene. The metabolites present in *C. longa* at higher concentrations include β-bisabolene, unidentified 5, unidentified 6, β-cedrene, cedr-9-ene, α-curcumene, curlone, β-elemenone, (Z)-β-farnesene, germacrene D, α-patchoulene, β-sesquiphellandrene, terpinolene, turmerone, ar-turmerone, unidentified 1 and unidentified 3. Several of these metabolites were major volatile components in essential oils and natural phytochemicals known to have biological activity. Potent anti-inflammatory, antioxidant, and antimicrobial activities are important functions of terpenoids such as bergamol [35], curcumene [36], turmerone [37,38], germacrene D [39] and caryophyllene [40]. *Curcuma* oil containing ar-turmerone, α,β-turmerone, and curlone has been used as an effective and safe antiplatelet agent against intravascular thrombosis [41].

**Table 1:** Compounds in the *Curcuma* extracts identified using GC/TOF MS.

	RT ^#^	Identified Metabolite	Formula	*Accurate Mass*	Main Fragments *m*/*z* (% bp) ^§^
1	5.98	Eucalyptol ^a^	C_10_H_18_O	154.1358	108.0945 (100); 154.1366 (96.7); 81.0707 (85.6)
2	6.81	Terpinolene ^†b^	C_10_H_16_	136.1252	121.1021 (100); 136.1261 (80.7); 93.0701 (80.2)
3	7.01	Bergamol ^c^	C_12_H_20_O_2_	196.1463	93.0704 (100); 69.0709 (44.0); 79.0534 (27.1)
I.S.-1	7.91	2,5-Dimethylphenol (IS)	C_8_H_10_O	122.0732	107.0501 (100); 122.0732 (95.6); 121.0664 (39.8)
4	12.44	δ-Elemene ^b^	C_15_H_24_	204.1878	121.1027 (100); 136.1266 (60.4); 93.0707 (55.8)
5	13.90	β-Elemene ^‡a^	C_15_H_24_	204.1878	93.0705 (100); 81.0710 (81.3); 147.1180 (68.7)
6	14.22	U-1	C_15_H_24_	204.1878	119.0862 (100); 93.0710 (92.0); 91.0545 (55.6)
7	14.78	β-Caryophyllene ^b^	C_15_H_24_	204.1878	133.1020 (100); 91.0545 (84.8); 93.0713 (83.0)
8	15.58	(Z)-β-Farnesene ^b^	C_15_H_24_	204.1878	69.0703 (100); 93.0712 (87.9); 133.1023 (59.6)
9	15.63	β-Cedrene ^c^	C_15_H_24_	204.1878	161.1329 (100); 69.0708 (86.3); 91.0546 (79.0)
10	15.77	α-Caryophyllene ^b^	C_15_H_24_	204.1878	93.0705 (100); 121.121.1014 (39.1); 80.0632 (26.9)
11	16.41	α-Curcumene ^b^	C_15_H_22_	202.1722	119.0865 (100); 132.0939 (82.1); 105.0709 (60.4)
12	16.47	Isoledene ^b^	C_15_H_24_	204.1878	161.1341 (100); 105.0706 (41.1); 204.1890 (31.0)
13		Germacrene D ^b^	C_15_H_24_	204.1878	161.1333 (100); 105.0705 (37.5); 91.0543 (31.3)
14	16.81	U-2	C_15_H_24_	204.1878	119.0844 (100); 93.0699 (82.3); 91.0544 (51.8)
15	17.15	β-Bisabolene ^a^	C_15_H_24_	204.1878	93.0701 (100); 69.0700 (76.99); 204.1880 (57.02)
16	17.62	β-Sesquiphellandrene ^b^	C_15_H_24_	204.1878	69.0707 (100); 91.0558 (98.5); 93.0713 (93.2)
17	17.68	α-Patchoulene ^c^	C_15_H_24_	204.1878	93.0706 (100); 107.0861 (98.7); 135.1187 (58.1)
18	19.08	β-Guaiene ^c^	C_15_H_24_	204.1878	161.1333 (100); 105.0709 (43.6); 204.1878 (36.1)
19	19.44	Cedr-9-ene ^b^	C_15_H_24_	204.1878	119.0863 (100); 93.0702 (64.2); 105.0707 (46.10)
20	20.1	U-3	C_15_H_24_	204.1878	119.0864 (100); 93.0703 (90.5); 91.0544 (70.3)
21	20.56	U-4	C_15_H_24_	204.1878	119.0868 (100); 93.0706 (81.1); 91.0544 (50.3)
22	21.23	Agaruspirol ^c^	C_15_H_26_O	222.1984	161.1336 (100); 204.1890 (98.1); 189.1658 (84.3)
23	21.43	ar-Turmerone ^b^	C_15_H_20_O	216.1514	119.0861 (100); 83.0494 (91.6); 216.1524 (49.3)
24	21.6	Turmerone ^b^	C_15_H_22_O	218.1671	105.0705 (100); 83.0499 (86.8); 120.0940 (59.0)
25	22.47	Curlone ^b^	C_15_H_22_O	218.1671	120.0937 (100); 83.0499 (27.5); 105.0705 (25.0)
26	22.81	U-5	C_15_H_24_O_2_	236.1787	180.1146 (100); 167.1069 (70.7); 109.0656 (35.6)
27	23.74	U-6	C_15_H_24_O_2_	2361791	180.1147 (100); 167.1079 (71.3); 109.0653 (33.4)
I.S.-2	34.93	Methyl nonadecanoate (IS)	C_20_H_40_O_2_	312.3028	74.0361 (100); 87.0444 (87.5); 312.2987 (86.9)

^#^ indicates the retention time of the compounds eluted using a DB-5 MS capillary column; ^†^ and ^‡^ represent metabolites verified using authentic reference compounds and chemical ionization mode, respectively; The compounds indicated by ^a,^ ^b,^ and ^c^ were identified with probabilities of 90%, 80%, and 70%, respectively; ^§^ *m*/*z*, relative intensity to base peak (% bp) shown in parenthesis. U-1 to U-6 refer to unidentified compounds **1** to **6**, respectively.

Terpenoids are the major phytochemicals in hexane extracts of *Curcuma*. Among the classes of terpenoids, monoterpenoids (C_10_), sesquiterpenoids (C_15_) and diterpenoids (C_20_) were detected in this study. The levels of these terpenoids in both *C. longa* and *C. aromatica* did not show clear differences between locations. However, we found clear differences in the levels of these terpenoids between *C. longa* and *C. aromatica*. Despite these distinct differences, unique patterns derived from classes of terpenoids were not observed. Based on these results, some classes of terpenoids, including C_10_, C_15_ and C_20_, were strongly affected by species variations. Previous studies reported that the levels of constituents of essential oils (non-polar extract) vary between *Curcuma* species [42], which is in agreement with our report.

### 2.2. Quantitative Analysis of Curcuminoids in Curcuma Species Using UPLC/Q-TOF MS

The high-resolution multiple reaction monitoring-like (MRM^HR^) workflow was used to selectively quantify curcuminoids within complex mixtures as it approaches an MRM-type experiment, providing a high-resolution full fragment ion spectra. This is termed MRM^HR^ because of the added high-resolution mass spectrometry (HRMS) selectivity of the product ion. After acquisition, high-resolution extracted ion chromatograms (XICs) of several fragment ions were generated and integrated in a similar fashion to the processing of triple-quadrupole MRM data. When using the HRMS-MRM scan (aka MRM^HR^) for small molecule quantitation, no MS method development is required [43].

**Figure 4:**
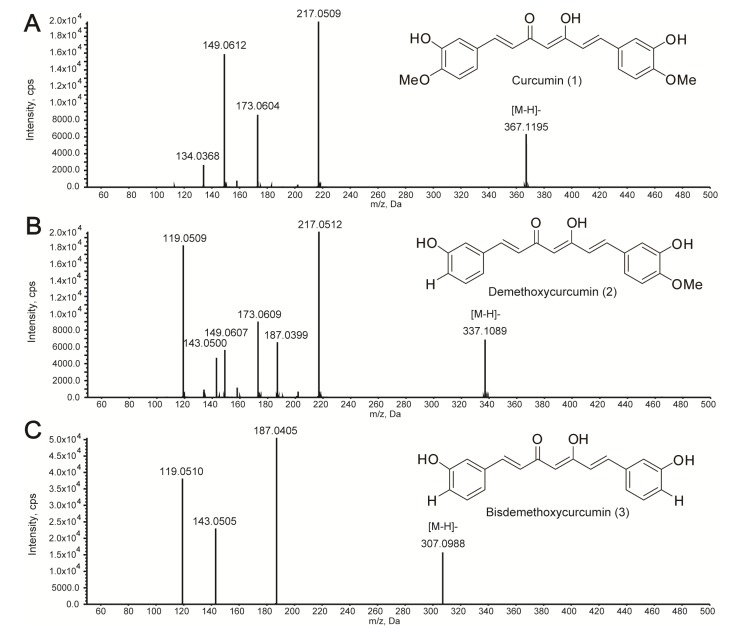
Spectra of curcuminoid compounds: (**A**) curcumin (**1**); (**B**) demethoxycurcumin (**2**); (**C**) bisdemethoxycurcumin (**3**).

Curcuminoids, including curcumin (**1**), demethoxycurcumin (**2**), and bisdemethoxycurcumin (**3**), are the major diarylheptanoid compounds present in *Curcuma* species, and many studies have attempted to identify and quantify these compounds [21,23,44]. Despite the relatively low sensitivity of (−)ESI compared to (+)ESI, negative mode (−)ESI-MS was effective in terms of identifying diarylheptanoids from turmeric. Diarylheptanoids can be detected by (−)ESI-MS because of the presence of phenolic hydroxyl groups, which enables these compounds to be easily ionized in (−)ESI mode [41]. The precursor ions of curcuminoids were detected as [M − H]^−^ at *m*/*z* 367.1195 for curcumin, 337.1089 for demethoxycurcumin, and 307.0988 for bisdemethoxycurcumin (Figure 4).

Quantification was performed using an external calibration curve with an internal standard, DSS (25 ng/mL), for instrument/analyst error correction. The relative response was calculated by dividing the area of each analyte by the area of the internal standard. Calibration curves, with concentrations ranging from 1.25 to 500 ng/mL, were generated using a combined standards solution (Table 2). The linear ranges used depended on the curcuminoid concentration in *Curcuma* extracts. Regression coefficients for calibration curves were at least 0.994 within this concentration range. The limit of quantification (LOQ) for curcuminoids was 0.5 ng/mL, which was also the lowest concentration in the calibration curve for demethoxycurcumin and bisdemethoxycurcumin.

The concentrations of curcuminoids in *Curcuma* extract, measured as described previously, are shown in Table 2. Of the curcuminoids, the curcumin concentration was highest in *Curcuma* extracts, while that of bisdemethoxycurcumin was lowest. The bisdemethoxycurcumin concentration in all *Curcuma* extracts was below the limit of quantification. Additionally, the demethoxycurcumin concentration in *C. aromatica* was uniformly higher than that in *C. longa*. This is not in agreement with a previous study in which the curcuminoid content of *C. longa* was higher than that of *C. aromatica* [45]. However, *Curcuma* species show variation in the contents of curcuminoids in rhizomes between varieties, locations, source, and cultivation conditions [24,46]. Because of this sensitivity to environmental variability, the curcuminoid contents of the *Curcuma* species used in this study may differ from previous reports. This result may be unique to *Curcuma* species grown in South Korea.

**Table 2:** Calibration parameters and curcuminoid concentrations in *Curcuma* extracts.

Samples	Calibration Parameters			*C. aromatica* Grown in Jeju-do		*C. aromatica* Grown in Jin-do		*C. longa* Grown in Jeju-do		*C. longa* Grown in Jin-do	
	Linear Range (ng/mL)	*R*^2^	^a^ LOQ (ng/mL)	Mean ± SE (μg/mg)	^b^ BQL (*n*)	Mean ± SE (μg/mg)	BQL (*n*)	Mean ± SE (μg/mg)	BQL(*n*)	Mean ± SE (μg/mg)	BQL (*n*)
Curcumin (**1**)	25–500	0.9989	0.5	1350.49 ± 72.66	0	239.24 ± 192.86	0	2591.43 ± 275.54	0	1240.38 ± 167.19	0
Demethoxycurcumin (**2**)	0.5–125	0.9999	0.5	276.60 ± 37.33	0	451.60 ± 26.79	0	15.49 ± 5.52	5	43.56 ± 0.00	9
Bisdemethoxycurcumin (**3**)	0.5–5	0.9944	0.5	-	10	-	10	-	10	-	10

^a^ LOQ: limit of quantification; ^b^ BQL: below the quantification limit.

The presence of curcuminoids in *Curcuma* extracts is an important determinant of bioactivity because curcuminoids, especially curcumin, possess a wide range of pharmacological activities. Although *C. longa* has ~10-fold more curcumin than *C. aromatica* [47], the total curcuminoid concentration differed only slightly between the two species in this study. A marked difference was observed between plants grown at different locations in terms of the curcumin concentration, the major compound in curcuminoids. Therefore, the curcuminoid content is affected by geographical region and species, although secondary metabolites are not affected by the growth environment.

## 3. Experimental Section

### 3.1. Chemicals and Reagents

Aceronitrile, hexane, and methanol (HPLC grade) were purchased from Burdick & Jackson (Muskegon, MI, USA). Distilled water was filtered using a Milli-Q Reagent Water System (Millipore, Billerica, MA, USA). Curcumin (96.4%), demethoxycurcumin (91.8%) and bisdemethoxycurcumin (91.8%) were obtained from Chromadex Inc. (Santa Ana, CA, USA). 2,5-Dimethylphenol, methyl nonadecanoic acid, sodium sulfate, and terpinolene were purchased from Sigma-Aldrich (St. Louis, MO, USA).

### 3.2. Plant Material

The rhizomes of *Curcuma aromatica* and *Curcuma longa* were obtained from the Jeju-do and Jin-do provinces of South Korea; 10 samples per subset were used to show significant differences between individuals. These 40 samples were cultivated under identical growth conditions in different regions from May to October 2010, and harvested from each region at the same time by the Korea Institute of Oriental Medicine. The rhizomes of *Curcuma* were chopped into small pieces, air-dried, and ground into powder. All dried samples were stored at −80 °C until required for GC- and LC-MS analysis.

The *Curcuma* species were identified morphologically based on the “Classification and Identification Committee of the Korea Institute of Oriental Medicine”, which is composed of nine experts in the fields of plant taxonomy, botany, pharmacognosy and herbalogy, and were confirmed by genomic profiling. Voucher specimens of plant materials were assigned the following accession numbers (*C. aromatica*-Jin-do KIOM201001003214-3216, *C. aromatica*-Jeju-do KIOM201001003311-3313, *C. longa*-Jin-do KIOM201001003217-3220, *C. longa*-Jeju-do KIOM201001003316-3319) and were preserved in the Herbarium of the Korea Institute of Oriental Medicine.

### 3.3. Procedures for Preparation of Sample Solution

#### 3.3.1. Procedures for Preparation of Sample Solution for GC/TOF MS Analysis

To extract non-polar metabolites, dried *Curcuma* rhizomes (50 mg) and internal standard solutions (500 μL) were transferred into 8-mL glass vials. Internal standard solutions were composed of methyl nonadecanoic acid (1 mg/mL) and 2,5-dimethylphenol (1 mg/mL) in hexane. After addition of methanol-water (2 mL, 1:1, v/v), the sample was vortexed for 1 min and sonicated for 20 min at 40 °C. To extract volatile metabolites, the non-polar solvent *n*-hexane (2 mL) was added and vortexed twice for 1 min each. The combined extracts were then placed at room temperature for 20 min, with the two phases separated by centrifugation at 2499 ×*g* for 5 min at 25 °C. The supernatant (non-polar phase) was transferred into a new glass vial and the remaining water was removed using anhydrous sodium sulfate. A micropipette, whose tip was fitted with a small piece of cotton, was used to filter out dust particles and impurities from the aliquot. A portion of the extract (100 μL) was diluted with *n*-hexane (100 μL) and transferred to a 2-mL GC vial with an infused insert.

#### 3.3.2. Procedures for Preparation of Sample Solution for UPLC/Q-TOF MS Analysis

Frozen *Curcuma* rhizome (100 mg) and sodium 2,2-dimethyl-2-silapentane-5-sulfonate (10 µL, 0.5 mg/mL) in methanol-water (1:1, v/v) (internal standard) were transferred to a 8-mL glass vial and methanol-water (1 mL, 1:1, v/v) was added. The sample was then mixed for 1 min, sonicated for 20 min at 40 °C, and centrifuged at 2499 ×*g* for 15 min at 25 °C. To remove any particles from the samples, crude supernatant was filtered using a 0.22-µm PTTE syringe filter (Millipore); the corresponding rhizome extracts (5 µL) were then individually diluted with methanol-water (995 μL, 1:1, v/v) and transferred to an autosampler vial.

### 3.4. Instrumental Analysis

#### 3.4.1. Instrumental Analysis of Non-Polar Extracts by GC/TOF MS

GC/TOF MS analysis was conducted using a 7890A gas chromatograph (Agilent Technologies, Palo Alto, CA, USA) equipped with a GCT premier TOF mass spectrometer (Waters, Manchester, UK). The DB-5 MS capillary column (30 m × 0.25-mm i.d., 0.25-µm film thickness, 5% diphenyl-95% dimethylsiloxane phase) was obtained from J&W Scientific (Folsom, CA, USA). The GC oven temperature began at 60 °C for 2 min and then increased to 100 °C at a rate of 20 °C/min and then to 220 °C at a rate of 4 °C/min. The final temperature of the program (300 °C) was attained at a rate of 25 °C/min and maintained for 5 min. Samples (1 µL) with a split ratio of 1:5 were injected. Helium was used as carrier gas at a constant flow rate of 1 mL/min. The temperature of the GC injector port and MS interface were set to 250 °C and 280 °C, respectively. MS acquisition conditions used were an electron impact (EI) ionization of 70 eV, a detector voltage of 2300 V, an inter-scan delay of 0.05 s, a scan time of 0.15 s, and a mass range of 50–800 atomic mass units (amu) at 5 spectra/s.

The GC/TOF MS raw data were analyzed using the MarkerLynx Applications Manager version 4.1 (Waters) for mass spectral peak identification and quantification with the following parameters: retention-time range, 5–36 min; mass range 50–600 Da; mass tolerance, 0.05; noise elimination level, 10.00; RT tolerance, 0.05 min. The internal standard (methyl nonadecanoic acid) was used for data normalization [19] and peak retention time alignment. The resulting matrix contains peak index (retention time-*m*/*z* pairs), sample names (observations), and ion intensity information (variables). The matrix was further reduced by removing any peaks with missing values (ion intensity = 0) in more than 80% of all samples and combining peaks within an RT window of 0.1 min. The ion peaks generated using the internal standard were also removed. Compounds were identified by comparing mass spectra with the National Institute of Standards and Technology mass spectral library (National Institute of Standards and Technology (NIST) MS search 2.0).

The resulting GC/TOF MS data matrices were imported into SIMCA-P version 12.0 (Umetrics, Umeå, Sweden) for chemometric analyses. Prior to multivariate analysis, all imported data sets were scaled using the unit variance (UV) scaling method, where each variable was divided by the standard deviation of the column values and centered, which provides base weight to the GC/TOF MS data set. Principal components analysis (PCA) was used to classify individual compounds within several reference groups. Partial least-squares discriminant analysis (PLS-DA) was used for multivariate pattern recognition analysis and supervised pattern recognition methods to examine intrinsic variation within the data set. The fit of the model to the data was described by *R*^2^ and *Q*^2^ values (where *R*^2^ describes the goodness of fit and *Q*^2^ indicates predictability).

Welch’s *t*-test was performed using Microsoft Office Excel 2007 (Microsoft Corporation, Alberquerque, NM, USA) to determine whether differences in metabolite levels among samples were significant. The differences were tested to a 95% probability level (*p* < 0.05). For multiple comparisons, Bonferroni’s corrections were applied and false discovery rates (FDRs) were computed from the overall *p*-values.

#### 3.4.2. Instrumental Analysis of Curcuminoids by UPLC/Q-TOF MS

Three curcuminoids (curcumin (**1**), demethoxycurcumin (**2**), and bisdemethoxycurcumin (**3**)) were analyzed using UPLC/Q-TOF MS. Separation of analytes was performed using a UPLC system (Acquity UPLC system; Waters) equipped with a ethylene bridged hybrid (BEH) C18 column that was 100 × 2.1 mm with a 1.7-µm particle size (Waters). The column temperature was set to 40 °C and the auto-sampler was maintained at 10 °C. The end sample (10 µL) was injected using the full-loop method. The mobile phase consisted of HPLC-grade water with 0.1% formic acid (A) and acetonitrile with 0.1% formic acid (B), and was eluted at 0.45 mL/min. The initial composition of mobile phase B was held at 5% for 1 min and increased to 95% after 6 min. To equilibrate the separation system, the mobile phase composition was reset to 5% for 3 min.

This UPLC system was connected to a triple TOF 5600 MS/MS system (AB Sciex, Framingham, MA, USA) equipped with an electrospray DuoSpray ion source (AB Sciex) operating in negative ion mode. The total ion chromatogram was acquired using the following operating parameters: a capillary voltage of −4500 V, a nebulizer pressure of 50 psi, a drying gas pressure of 50 psi, a curtain gas pressure of 30 psi, a source temperature of 500 °C, a declustering potential of −70 eV, and a collision energy of −10 eV. To obtain reliable results, specific values of collision energy (CE) were used. The CE for curcumin (**1**), demethoxycurcumin (**2**), bisdemethoxycurcumin (**3**), and the internal standard (DSS) were set at −20, −20, −18, and −30 eV, respectively, with an accumulation time of 0.1 s. Mass spectra were acquired from *m*/*z* 50 to *m*/*z* 500 for 10 min. Accurate mass measurements of each peak were obtained using an automated calibrant delivery system (CDS) with 0.2 mL/min of a negative APCI calibrating solution (AB Sciex) containing several internal reference masses (*m*/*z* 145, *m*/*z* 265, *m*/*z* 278, *m*/*z* 353, *m*/*z* 404, *m*/*z* 441, *m*/*z* 617, and *m*/*z* 793).

Stock solutions containing curcuminoids (curcumin (**1**), demethoxycurcumin (**2**), and bisdemethoxycurcumin (**3**)) and an internal standard (DSS) of varying concentrations were used to construct calibration curves. Each concentration of the mixed standard solution was injected in duplicate, and a calibration curve was constructed by plotting the peak area/internal standard peak ratio *versus* the concentration of each analyte using the MultiQuent™ software, version 2.1 (AB Sciex, Framingham, MA, USA).

## 4. Conclusions

In summary, we conducted metabolite profiling of two *Curcuma* species using GC and LC-MS to examine differences between species and geographical location. The use of GC and LC-MS allows for analysis of both terpenoids and curcuminoids in *Curcuma* extracts. GC/TOF MS coupled with multivariate analysis showed that terpenoids are important markers for characterizing the two *Curcuma* species. Target metabolite profiling of curcuminoids using UPLC/Q-TOF MS showed that the content of curcuminoids within plants are affected by geographical region as well as species. Significant differences in terpenoids and curcuminoids indicate that different *Curcuma* species or *Curcuma* grown in different locations may exhibit different biological properties. In addition, our study suggests that metabolite profiling, combined with GC and LC-MS, is a useful tool for analysis of the chemical composition of *Curcuma* species grown at different locations.

## Supplementary Materials

VIP value, *p*-value, and *q*-value of 63 significantly different features between *C. longa* and *C. aromatica* obtained from the GC/TOF MS spectra (Table S1). Supplementary materials can be accessed at: http://www.mdpi.com/1420-3049/19/7/9535/s1.
