# Supplementary material for: Secondary Metabolite Profiling of Curcuma Species Grown at Different Locations Using GC/TOF and UPLC/Q-TOF MS
Source: Molecules. 2014 Jul 4;19(7):9535–51. doi: 10.3390/molecules19079535 (PMC6270825; doi:10.3390/molecules19079535)

Supplementary Data

Table S1. VIP value, *p*-value and *q*-value.

**Sample ID** [a]	**VIP Value** [b]	***p*-value** [c]	***q*-value** [d]
Unidentified 2	1.46871	4.6×10^{-14}	2.71×10^{-13}
105	1.4668	5.76×10^{-14}	2.71×10^{-13}
β-Sesquiphellandrene	1.46006	1.24×10^{-13}	4.17×10^{-13}
(Z)-β-Farnesene	1.45881	6.05×10^{-14}	2.71×10^{-13}
β-Bisabolene	1.45538	2.67×10^{-13}	5.98×10^{-13}
Cedr-9-ene	1.44809	2.46×10^{-13}	5.79×10^{-13}
85	1.44505	3.37×10^{-13}	6.48×10^{-13}
Unidentified 1	1.4446	6.2×10^{-13}	9.26×10^{-13}
Unidentified 4	1.44443	5.63×10^{-13}	8.81×10^{-13}
100	1.40925	1.41×10^{-11}	1.56×10^{-11}
64	1.40672	1.94×10^{-11}	1.94×10^{-11}
Germacrene D	1.40564	2.11×10^{-11}	2.04×10^{-11}
60	1.40451	2.16×10^{-11}	2.08×10^{-11}
108	1.40317	7.39×10^{-12}	9.36×10^{-12}
66	1.4022	2.63×10^{-11}	2.36×10^{-11}
α-Patchoulene	1.399	3.51×10^{-11}	2.85×10^{-11}
Curlone	1.39647	3.99×10^{-11}	3.08×10^{-11}
98	1.39445	4.33×10^{-11}	3.23×10^{-11}
Turmerone	1.39171	5.86×10^{-11}	3.94×10^{-11}
Unidentified 3	1.39008	5.52×10^{-11}	3.8×10^{-11}
β-Cedrene	1.3655	3.43×10^{-10}	2.17×10^{-10}
44	1.36149	2.26×10^{-08}	1.13×10^{-08}
Unidentified 5	1.35678	5.04×10^{-10}	3.08×10^{-10}
15	1.35545	1.96×10^{-09}	1.1×10^{-09}
Unidentified 6	1.344	1.36×10^{-09}	7.91×10^{-10}
13	1.29387	1.46×10^{-05}	4.19×10^{-06}
21	1.29057	1.68×10^{-08}	8.64×10^{-09}
49	1.26715	1.48×10^{-07}	5.97×10^{-08}
68	1.25944	5.4×10^{-08}	2.5×10^{-08}
110	1.25247	4.78×10^{-08}	2.25×10^{-08}
β-Guaiene	1.24747	1.06×10^{-07}	4.47×10^{-08}
67	1.24268	4.6×10^{-07}	1.67×10^{-07}
10	1.23714	1.48×10^{-08}	7.67×10^{-09}
α-Curcumene	1.23347	1.08×10^{-07}	4.54×10^{-08}
114	1.21633	1.7×10^{-07}	6.73×10^{-08}
95	1.2137	4.08×10^{-07}	1.5×10^{-07}
Terpinolene	1.20998	1.04×10^{-07}	4.4×10^{-08}
Isoledene	1.19519	7.98×10^{-07}	2.75×10^{-07}
ar-Turmerone	1.18797	4.47×10^{-07}	1.63×10^{-07}
78	1.1791	5.47×10^{-07}	1.93×10^{-07}
59	1.16759	2.18×10^{-06}	7.03×10^{-07}
α-Caryophyllene	1.1616	2.25×10^{-06}	7.24×10^{-07}
30	1.15761	2.36×10^{-06}	7.57×10^{-07}
Bergamol	1.13519	5.85×10^{-06}	1.79×10^{-06}

Table S1. *Cont.*

Sample ID [a]	VIP Value [b]	*p*-value [c]	*q*-value [d]
61	1.11202	2.7×10^{-05}	7.13×10^{-06}
Agaruspirol	1.11154	5.97×10^{-06}	1.83×10^{-06}
115	1.11091	1.22×10^{-02}	1.94×10^{-03}
β-Caryophyllen	1.09192	1.5×10^{-05}	4.3×10^{-06}
β-Elemene	1.06527	3.29×10^{-05}	8.39×10^{-06}
9	1.06218	3.21×10^{-05}	8.22×10^{-06}
δ-Elemene	1.05759	3.94×10^{-05}	9.65×10^{-06}
19	1.05059	6.54×10^{-05}	1.55×10^{-05}
35	1.04962	9.44×10^{-05}	2.17×10^{-05}
51	1.04929	2.04×10^{-05}	5.64×10^{-06}
22	1.04783	6.67×10^{-05}	1.57×10^{-05}
62	1.04369	2.06×10^{-05}	5.67×10^{-06}
89	1.03099	2.26×10^{-05}	6.14×10^{-06}
17	1.02114	3.35×10^{-05}	8.51×10^{-06}
92	0.989471	1.48×10^{-04}	3.31×10^{-05}
74	0.977628	3.97×10^{-05}	9.71×10^{-06}
55	0.950677	1.04×10^{-04}	2.38×10^{-05}
63	0.928019	2.31×10^{-04}	5.02×10^{-05}
Eucalyptol	0.922692	9.02×10^{-04}	1.77×10^{-04}

[a] Sample ID means arbitrary-numbered ID of 121 features obtained from GC/TOF MS spectrum data; [b] Variable importance in the projection (VIP) was obtained by Partial Least Squares Discriminant analysis (PLS-DA) with threshold of 0.9; [c] *p*-value was calculated using the Welch's *t*-test with significance at $p < 0.05$; [d] False discovery rates (FDRs, *q*-value) were calculated from the overall *p*-values.

Figure S1. Permutation analysis of PLS-DA model derived from two *Curcuma* species. Statistical validation of the PLS-DA model was performde by permutation analysis using 100 permutations.

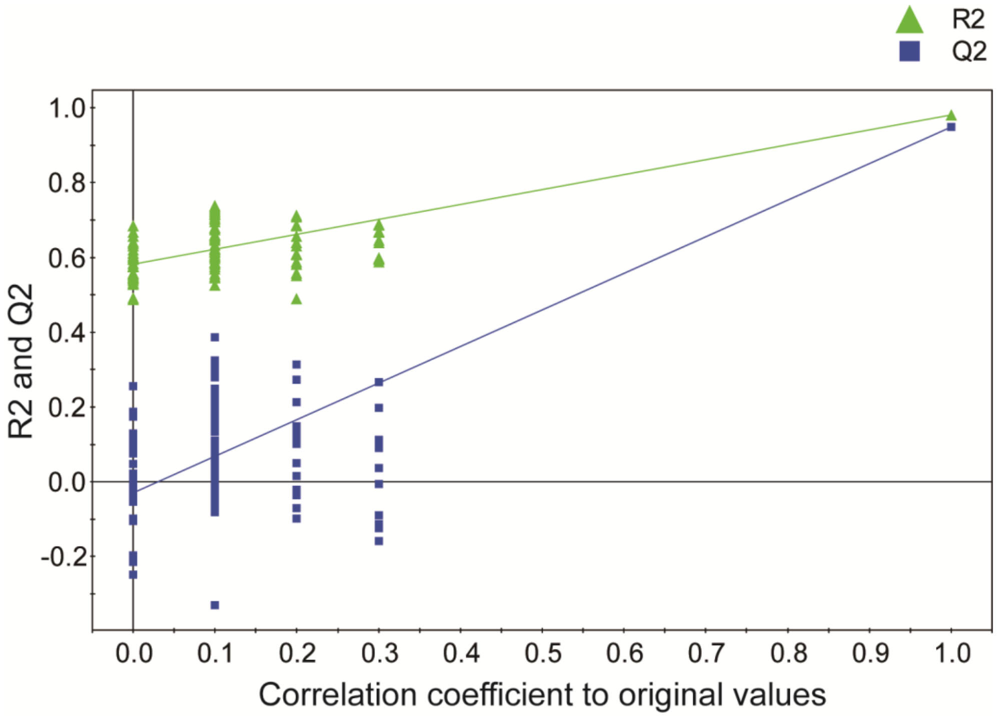

Supplement: Supplementary file 1 [file molecules-19-09535-s001.pdf]
